# Investigation of iron uptake and virulence gene factors (*fur, tonB, exbD, exbB, hgbA, hgbB1, hgbB2* and *tbpA*) among isolates of *Pasteurella multocida* from Iran

**Published:** 2019-06

**Authors:** Motahare Feizabadi Farahani, Majid Esmaelizad, Ahmad Reza Jabbari

**Affiliations:** 1 Department of Central Laboratory, Razi Vaccine and Serum Research Institute, Agricultural Research, Education and Extension Organization, Tehran, Iran; 2 Department of Pasteurella National Research Laboratory, Razi Vaccine and Serum Research Institute, Agricultural Research, Education and Extension Organization, Tehran, Iran

**Keywords:** *Pasteurella multocida*, Virulence factors, Iron acquisition

## Abstract

**Background and Objectives:**

Iron is an essential compound in metabolic pathway of wide range of organisms. Because of limited free iron supply in mammalian and avian hosts, bacteria have applied various ways to acquire iron.

**Materials and Methods:**

In this study, the frequency of 8 iron acquisition factors was examined among 63 avian and ovine *Pasteurella multocida* field isolates and their vaccine strains using PCR method.

**Results:**

Five candidate genes (*fur, tonB, exbD, exbB* and *hgbA*) were identified among all isolates. For the first time, 2 loci (*hgbB1* and *hgbB2*) of the *hgbB* gene were identified, which were previously reported as 1 gene. Also, it was found that 5 ovine and 1 avian isolates possessed all the virulence factors, which could also be considered for evaluating the frequency of other virulence factors.

**Conclusion:**

More studies need to be conducted on the frequency of all other virulence factors among these isolates, which can provide basic information for improvement or substitution of current vaccinal strains. Overall, as the new designed sets of primers showed more potential in detecting the corresponded genes, researchers can consider them in further studies.

## INTRODUCTION

*Pasteurella multocida* (*P. multocida*) is a Gram-negative bacterium that belongs to the *Pasteurellaceae* family (1, 2). It is a nonmotile, facultive anaerobic microbe which can be isolated from a variety of species, including domesticated mammals and poultry throughout the world (3, 4). *P. multocida* causes atrophic rhinitis in swine, snuffle in rabbits, fowl cholera among avian, haemorrhagic septicaemia in bovine, and enzootic bronchopneumonia in both bovine and ovine herds (2, 5). There are 5 capsular serogroups: A, B, D, E and F; and according to lipopolysaccharide antigen expression, they are arranged into 16 somatic serotypes (6).

Iron is a crucial compound for most organisms, due to its essential function in metabolic transport chains (3). Bacteria apply various systems to acquire iron such as siderophores or outer membrane proteins (7). Siderophores are iron ligands which compete with protein carriers to bind to ferric iron, while some outer membrane proteins can also acquire iron from the host Fe-binding molecules, such as lactoferrin, heme, ferritin, hemoglobin, and transferrin (4, 8).

Various virulence factors (VF) found in *P. multocida* include proteins with functions associated with adherence and colonization (PtfA, FimA, Hsf-1, Hsf-2, Pf hA and TadD), iron acquisition proteins (ExbB, ExbD, TonB, HgbA, HgbB and Fur), extracellular enzymes such as neuraminidases (NanB and NanH) and superoxide dismutases (Soda, SodC and TbpA), hyaluronidase (PmHAS), toxins (ToxA), lipopolysaccharides (LPS), capsular and outer membrane proteins (OmpA, Omph, Oma87 and PlpB) (9, 10).

Numerous crucial functions have been reported about the correlation between adhesion proteins and bacterial virulence, provoking adhesion and colonization. Sialic acid can be found in a conjugated form to glycolipids and glycoproteins of eukaryotic cells. The presence of sialidases enzymes is crucial in removing these compounds. Additionally, OmpH and PlpE are protective antigens which have been detected in A:1, A:3, and A:4 serotypes from poultry suffered from fowl cholera and cattle with shipping fever (10).

Gram-negative bacteria can apply 2 different types of hemoglobin receptor proteins to boost the transmission of either iron or heme group into the cell. Some extract and deliver the heme group into the cell from hemoglobin by secreting a hemoglobin-binding protein while the other use a specific receptor which can interact with haemoglobin directly and facilitate the transmission of both iron and heme group. Overall, transmission of either iron or heme across the outer membrane by all of the mentioned mechanisms requires the expression of the tonB, exbD and exbB genes (7).

TonB induces the transfer of energy required for transportation of iron into bacterial cells by linking both cytoplasmic and outer membranes of the bacteria. Presence of ExbD and ExbB proteins, as the inner membrane stabilizer for TonB protein, is essential for this process (11).

Regulation of gene expression associated with iron is generally controlled by *fur* gene in Gram-negative bacteria (12). When iron is abundant, *fur* interacts with ferrous iron and binds to the conserved promoter regions known as Fur box and inhibits gene transcription (13).

HgbA and HgbB are 2 proteins that are used by *P. multocida* to acquire iron directly from the haem component. HgbA gene is reported to be distributed more regularly among isolates, whereas *hgbB* gene prevalence varies among strains according to the host origin and the animal disease status. Another protein known as TbpA, a transferrin binding protein, is reported as an epidemiological marker among cattle and plays an essential role in extraction of iron from transferrin (2).

Few studies have investigated the association between various hosts and frequency of the mentioned VFs among *P. multocida* isolates which can significantly increase the current knowledge about its pathogenicity, epidemiology, and development of current vaccinal strains (14, 10). Thus, findings of this study can shed light on correlation between the host and frequency of various iron uptake VFs among Iranian *P. multocida* isolates from different regions and the efficiency of current avian and ovine vaccinal strains on recent field isolates.

## MATERIALS AND METHODS

### Bacterial samples.

A total of 63 lyophilized *P. multocida* isolates (30 avian and 33 ovine isolates, available in *Pasteurella* National Research Laboratory at Razi Vaccine and Serum Research Institute) from different provinces of Iran, were used to investigate the presence and prevalence of 8 iron virulence factor genes (*fur, tonB, exbD, exbB, hgbA, hgbB1, hgbB2, tbpA*). The frequency of *hgbB* amplicon was also investigated among the isolates. The information related to origin, host, and serotype of each avian isolates are shown in Table 1, but the related information about the ovine isolates was not available.

**Table 1.** Classification avian *Pasteurella multocida* isolates based on host, origin and serotype

**Isolate ID**	**Host**	**Province**	**County**	**Serotype**
PM01	Chicken	Gilan	Astara	1
PM02	Chicken	Mazandaran	Sari	3×4
PM03	Chicken	Mazandaran	Sari	4
PM04	Chicken	Mazandaran	Ghaemshahr	1
PM05	Chicken	Gilan Gilan	Rasht	1
PM06	Chicken	Mazandaran	Rasht	1
PM07	Chicken	Mazandaran	Ghaemshahr	3×4
PM08	Chicken	Gilan	Sari	1
PM09	Chicken	Gilan	Talesh	1
PM10	Chicken	Mazandaran	Talesh	2
PM11	Duck	Gilan	Amol	3
PM12	Duck	Gilan	Shaft	4
PM13	Chicken	Mazandaran	Talesh	-
PM14	Duck	Mazandaran	Amol	3
PM15	Chicken	Mazandaran	Amol	4
PM16	Chicken	Mazandaran	Sari	1
PM17	Duck	Mazandaran	Ghaemshahr	1
PM18	Chicken	Mazandaran	Amol	1
PM19	Duck	Gilan	Sari	1
PM20	Chicken	Gilan	Talesh	3
PM21	Duck	Tehran	Rasht	1
PM22	Chicken	Tehran	Tehran	1
PM23	Chicken	Tehran	Tehran	1
PM24	Chicken	Tehran	Tehran	1
PM25	Chicken	Tehran	Tehran	1
PM26	Chicken	Tehran	Tehran	1
PM27	Chicken	Zanjan	Tehran	1
PM28	Chicken	Tehran	Zanjan	1
PM29	Chicken	Gilan	Tehran	1
PM30	Chicken		Rasht	1

### DNA extraction and concentration.

Genomic DNA was extracted via boiling method from a 24-hour blood agar culture. The process included 1mL of each fresh blood agar culture dissolved in 1 mL of 1× TE buffer made up of 10 mM of Tris-HCl (PH 8) and 1 mM of EDTA; then, they were put in boiled water (water bath) for 15 minutes. The samples were centrifuged for 5 minutes at 10000 ×g. Supernatant, which contained DNA, was used for PCR tests (15).

The quality and quantity of the extracted DNA was evaluated at the OD of 260–280 nm using a spectro-photometer (Eppendrof, Germany) (14).

### Conventional PCR program.

The primers’ sequences and the ampliqon size of PCR products are shown in Table 2.

**Table 2.** Iron acquisition virulence factors, their function, primer sequence, amplicon size, and the source of each primer pair

**Primers**	**Description**	**Primer sequence (5′-3′)**	**Amplicon Size (bp)**	**Reference**
(KMT1) pm	Identification all *Pasteurella multocida*	ATCCGCTATTTACCCAGTGG GCTGTAAACGAACTCGCCAC	460	11
CAPA	Capsule protein A	TGCCAAAATCGCAGTCAG TTGCCATCATTGTCAGTG	1044	11
CAPB	Capsule protein B	CATTTATCCAAGCTCCACC GCCCGAGAGTTTCAATCC	758	11
Fur1	Ferricuptake regulation	GTTTACCGTGTATTAGACCA CATTACTACATTTGCCATAC	244	7
Fur2	Ferric uptake regulation	AAAGCGGGGYTGAAAATYAC CGCATTTCTTGAYATYRCTACATTRC	390	This study
TonB	Iron acquisition	CGACGGTGAAACCTGAGCCA CCGAGCGATAAGCATTGACT	261	7
TonB-exbBD	Iron acquisition	GGTGGTGATATTGATGCGGC GCATCATGCGTGCACGGTT	1144	5
HgbA	Hemoglobin-binding protein	TGGCGGATAGTCATCAAG CCAAAGAACCACTACCCA	419	5
HgbB	Hemoglobin-binding protein	ACCGCGTTGGAATTATGATTG CATTGAGTACGGCTTGACAT	788	5
HgbB1	Hemoglobin-binding protein	CCGTTTCACTTTTGCTCTGGATA GGAGTGCTCCTTATAATGAAGA	441	This study
HgbB2	Hemoglobin-binding protein	CCGTTTCAGCGTGGCATTGGATG AGTATGCTCACGATAGTGTGTG	441	This study
TbpA	Transferrin-binding protein	ACAACGTTCTGCTCTCCAG CCTTTGCTGTAGCTCCCTTG	899	This study

The PCR program for every primer pair is shown in Table 3. All reactions were done in 20 μL of the total volume. For all reactions, 1 μL of DNA template was added to the 10 μL, Master Mix (Ampliqonco.), with 7 μL of double-distilled water, and 1 μL from 10 pmol of each primer pair. Specific *P. multocida* PCR (*kmt1* gene) and isolates’ capsular type were investigated using approved primer sets and PCR protocols (16).

**Table 3.** PCR conditions for Iron virulence factors

**Primers**	**Initial denaturation (°C / sec)**	**Denaturation (°C/sec)**	**Annealing (°C/ sec)**	**Extension (°C/ sec)**	**Final extention (°C/ min)**	**No. of complete cycles**
KMT1	94°C, 3 min	94°C, 1 min	51°C, 30 s	72°C, 45 s	72°C, 5 min	35
CAPA, CAPB	93°C, 3 min	93°C, 1 min	60°C, 30 s	72°C, 30 s	72°C, 5 min	35
Fur1	93°C, 3 min	93°C, 1 min	51°C, 20 s	72°C, 20 s	72°C, 5 min	35
Fur2	93°C, 3 min	93°C, 1 min	52°C, 30 s	72°C, 40 s	72°C, 5 min	35
TonB	93°C, 3 min	93°C, 1 min	54 °C, 30 s	72°C, 30 s	72°C, 5 min	35
TonB-exbBD	93°C, 3 min	93°C, 1 min	52°C, 30 s	72°C, 1 min	72°C, 5 min	35
HgbB	93°C, 3 min	93°C, 1 min	52°C, 30 s	72°C, 40 s	72°C, 5 min	35–37
HgbA, HgbB1, HgbB2	93°C, 3 min	93°C, 1 min	52°C, 30 s	72°C, 40 s	72°C, 5 min	35–38
TbpA	93°C, 3 min	93°C, 1 min	52°C, 30 s	72°C, 1 min	72°C, 5 min	35–38

### Primer designing.

All new sets of primers were designed based on all existed sequences in GeneBank. The first set of primers for detecting *fur* gene (fur1), used by previous researchers, were aligned in NCBI site (6, 7). Primer sequence was investigated among all the samples that belonged to *P. multocida*. In forward primer sequence, the 16^th^ nucleotide, which was reported as G, was found as A nucleotide among some reported nucleotide sequences; and in reverse primer sequence, the last 3′ nucleotide, C, was reported as A nucleotide. These SNPs decreased the covering of target sequences and could increase false negative results.

In this study, 2 specific sets of primers were designed that could specifically detect *hgbB* gene, with the desired amplicon size of 441 bp.

### Electrophoresis.

The electrophoresis of PCR products was performed in 1% agarose gel and stained with ethidium bromide. The frequency of each gene is reported in Table 4. The approved PCR products, which confirmed the efficiency of new sets of primers, are shown in Table 5.

**Table 4.** Frequency of the evaluated iron uptake VFs in this study among Iranian avian and ovine isolates

**Reference**	**Host**	**Genes**											**Capsular type**	**Origin**

		***fur1***	***fur2***	***hgbA***	***hgbB***	***hgbB1***	***hgbB2***	***exbD***	***tonB***	***exbB***	***exbBD-TonB***	***tbpA***		
This study	Avian	93.3	100	100	100	36.66	80	100	100	100	100	23.3	A	Iran
This study	Ovine	93.74	100	100	75	54.54	27.27	100	100	100	100	100	A	Iran

**Table 5.** Classification of 30 isolates of *P. multocida* from poultry based on the prevalence of iron virulence factors

**Group**	**Absent genes**	**Isolate name**	**Number**	**Percentage**
I	HgbB2-	PM30	1	3/3%
II	HgbB2-, TbpA-	PM28, PM26, PM22, PM06	4	13/3%
III	HgbB1-	PM29, PM21, PM20, PM17, PM08	5	16/6%
IV	TbpA-	PM25, PM23, PM15, PM07, PM03	5	16/6%
V	All Positive	PM04	1	3/3%
VI	HgbB1-,TbpA-	PM27, PM24, PM19, PM18, PM16, PM14, PM13, PM12, PM11, PM09, PM05, PM02, PM01	13	43/3%
VII	HgbB1-, HgbB2-, TbpA-	PM10	1	3/3%

### Data analysis.

Matrix 0–1 was designed based on the presence or absence of 8 iron virulence factor genes (*fur, tonB, exbD, exbB, hgbA, hgbB1, hgbB2, tbpA*).

Data were analysed by DendroUPGMA software (2002) and are shown in Tables 5 and 6.

**Table 6.** Classification of 33 ovine *P. multocida* isolates according to the prevalence of iron virulence factors investigated in this study

**Group**	**Absent genes**	**Isolate name**	**Number**	**Percentage**
I	All positive	PM30, PM27, PM22, PM06, PM08	5	15.15
II	hgbB2-	PM32, PM31, PM29, PM28, PM26, PM17, PM16, PM15, PM12, PM11, PM05, PM07	12	36.36
III	hgbB1-	PM23, PM21, PM18, PM09	4	12.12
IV	hgbB1-, hgbB2-	PM25, PM24, PM20, PMb2, PM19, PM14, PM13, PM04, PM03, PM02, PM01	11	33.33

## RESULTS

### Capsular typing output.

The specific PCR test based on *kmt1* gene was positive for all 63 samples and demonstrated that 30 avian and 33 ovine *P. multocida* isolates belonged to capsular type A.

### Conventional PCR results.

Based on the new designed primer pair in this study, the *fur* gene was present in all the studied isolates.

The designed primers (*hgbB1* and *hgbB2*) could amplify these loci in *P. multocida* full genomes. Among the studied collection, the *hgbB* gene was present in 100% of avian isolates, while 75% of ovine isolates showed the presence of *hgbB* gene.

The results indicated that 36.6% of avian and 54.54% of ovine isolates possessed locus 1. Additionally, locus 2 was present in 80% of avian and 27.27% of ovine isolates. Both loci were present in 20% of avian and 15.15% of ovine isolates. Also, 16.66% of poultry and 39.39% of ovine isolates contained locus 1, while locus 2 was present in 60% and 12.12% of avian and ovine isolates, respectively.

With the exception of *tbpA*, the *fur, hgbA, exbB, exbD* and *tonB* genes were present in all avian and ovine isolates. Moreover, *tbpA* was detected in all ovine isolates, whereas only 23.3% of avian isolates possessed this gene (Table 4).

### Confirmation of new primer pairs potential.

The PCR products of new sets of primers were sequenced and aligned with all present sequences of *P. multocida* in GeneBank. The *fur* gene has been submitted with KX832975.1, KX832974.1 accession numbers and KX781178.1, KX781177.1, KX781176.1 are related to *tbpA* gene.

### Dendrogram analysis.

Based on the presence of *fur2, tonB, exbB, exbD, hgbA, tbpA* gene, and *hgbB1* and *hgbB2* loci, the avian and ovine isolates were clustered into 7 and 4 groups, respectively. Moreover, 15.15% of ovine and 3.3% of avian isolates possessed all the VFs in this study. The determinant factors in grouping the avian and ovine isolates concerning the iron uptake VFs were *tbpA, hgbB1* and *hgbB2* loci, while all other VFs were present in all isolates. Eventually, avian vaccine strain covered 43.3% of all avian isolates, while the bovine vaccine strain could only cover 33.33% of all ovine field isolates (Tables 5, 6).

## DISCUSSION

Epidemiological investigations on frequency of bacterial pathogens can help discover the origin of related diseases, which can further provide essential information for applying more efficient control measures (2).

Only few studies have previously investigated the prevalence of *hgbB* gene among *P. multocida* isolates. The primer pair, which was used by most researchers, could amplify both loci of this gene in *P. multocida* full genomes (*hgbB*). For the first time, in this study, the prevalence of each copy among avian and ovine isolates was examined using specific sets of primers (*hgbB1* for locus1 and *hgbB2* for locus 2). The *hgbB* gene was present in 100% of avian isolates, while 75% of ovine isolates showed the presence of this gene. Also, the presence of *hgbB* gene among avian isolates has been reported to be 100% in Iranian and Brazilian, 85% in German, and 76.9% in Indian isolates. Moreover, it has been demonstrated that 100% of Iranian and Indian cattle isolates possessed this gene, while some other studies indicated the presence of this gene in 57.4, 57.7, and 61.3% of ovine and cattle isolates from Iran, Germany, and Japan, respectively (2, 5, 9, 17–20). In contrast, it has been reported that only 40.9% of Iranian cattle and buffalo isolates from Khuzestan possessed this gene (10).

HgbA was present in 100% of ovine and avian isolates investigated in this study. The frequency of this gene among different hosts, irrespective of their origin, has been reported from 84.6% to 100%, while it was present in 73.9% of Brazilian rabbit isolates (2, 5, 6, 7, 17, 19, 20). In addition, a previous study on Iranian cattle and buffalo isolates indicated the presence of *hgbA* gene among 77.2% of all studied isolates (10).

The presence of *fur* gene in 93.3% of avian and 93.74% of ovine isolates was detected by previous published primers (Fur1). Sequence alignment of *fur* gene observed 2 SNPs in *fur1* primer positions. In this study, 2 primer pairs were used to investigate the frequency of *fur* gene among isolates. Using a new primer set (*fur2*), the positive results were increased to 100% in comparison to the previously published primer (Fur1). Meanwhile fur1 primer covered 82.6% of Iranian cattle isolates (9). Additionally, 96.7% of swine isolates that belonged to China possessed this gene (6). Ferreira (2012) found that the frequency of *fur* gene was only 4.3% among Brazilian rabbit isolates (20). Also, *tonB, exbB* and *exbD* genes are transcribed independently while are physically linked. The iron carrier components (transferrin, siderophore, haemoglobin) possess a conserved region known as *tonB* box, which is responsible for interaction with TonB protein. Binding of TonB box to the outer membrane receptor induces a change in TonB protein conformation and passes iron through TonB pore (11). The expression level of *tonB, exbD* and *exbB* alternation in response to low iron condition has been reported to be 2.5, 2.3 and 4.7 folds (3).

Furthermore, *tonB, exbB* and *exbD* were present among all ovine and avian isolates investigated in this study. Previous research showed the presence of *tonB* gene to be between 94.6% and 100% among *P. multocida* isolates from different regions and hosts. Also, *exbD* and *exbB* genes were frequency reported to be between 98.9%–100% and 82%–100%, respectively (5, 6, 9, 19). Also, tonB-exbBD primer set could amplify all 3 *tonB, exbB* and *exbD* genes, which has been detected among 60.8% of Brazilian rabbit isolates (20). Results of this study demonstrated that the frequency of tonB-exbBD was 100% among studied isolates. Previous research indicated that exbBD-tonB amplicon was also present in a high rate (90.9%) among buffalo and cattle isolates from Khuzestan province in Iran (10). TbpA is an iron-binding protein, which interacts directly with host iron-loaded glycoproteins and is expressed on the outer membrane of the bacterial cell (21). In this study, *tbpA* gene was present in 23.3% of poultry and 100% of ovine isolates. Previous researches reported the presence of this gene in 69.2% of poultry isolates from India (2). However, none of the avian isolates from Germany possessed this gene (5). The presence of this gene among cattle and sheep isolates was reported to be between 69%–100% in different regions. Also, 8.6% of rabbit isolates from Brazil contained this gene (18–20).

Moreover, new primer pairs which were designed in this study, for *fur*, 2 loci of *hgbB* gene (*hgbB1, hgbB2*) and *tbpA* genes, indicated more efficiency in detecting these genes among Iranian avian and ovine isolates. It is suggested that these primers can be used to ensure a precise detection of the target genes in further studies. Moreover, not any specific relationship between the frequency of investigated VFs and host and/or geography was observed among avian isolates. It has been stated that a suitable vaccine strain should contain the most virulence and immunogenic factors. In fact, the results of this study indicated that the current avian and bovine strains could not cover all the iron uptake VFs, while there are some avian (PM04) and ovine (PM30, PM27, PM22, PM06, and PM08) field isolates which demonstrated 100% coverage. The comparison between the vaccine strains and field isolates need to be investigated and updated continuously. Therefore, the results of this study, in addition to evaluating the presence of other VFs, can provide basic information for substituting current vaccines or choosing suitable isolates for polyvalent or recombinant vaccine strains in future.
